# Determination of PD-L1 Expression in Circulating Tumor Cells of NSCLC Patients and Correlation with Response to PD-1/PD-L1 Inhibitors

**DOI:** 10.3390/cancers11060835

**Published:** 2019-06-17

**Authors:** Melanie Janning, Franca Kobus, Anna Babayan, Harriet Wikman, Janna-Lisa Velthaus, Sonja Bergmann, Stefanie Schatz, Markus Falk, Lars-Arne Berger, Lisa-Marie Böttcher, Sarina Päsler, Tobias M. Gorges, Linda O’Flaherty, Claudia Hille, Simon A. Joosse, Ronald Simon, Markus Tiemann, Carsten Bokemeyer, Martin Reck, Sabine Riethdorf, Klaus Pantel, Sonja Loges

**Affiliations:** 1 Department of Oncology, Hematology and Bone Marrow Transplantation with section Pneumology, Hubertus Wald University Comprehensive Cancer Center Hamburg, University Medical Center Hamburg-Eppendorf, 20246 Hamburg, Germany; m.janning@uke.de (M.J.); francakobus@yahoo.de (F.K.); j.velthaus@uke.de (J.-L.V.); LarsArne.Berger@hopa.de (L.-A.B.); lisamarie.boettcher@googlemail.com (L.-M.B.); s.paesler@uke.de (S.P.); c.bokemeyer@uke.de (C.B.); 2 Department of Tumor Biology, Center of Experimental Medicine, University Medical Center Hamburg-Eppendorf, 20246 Hamburg, Germany; a.babayan@uke.de (A.B.); h.wikman@uke.de (H.W.); so.bergmann@uke.de (S.B.); tobias.gorges@astrazeneca.com (T.M.G.); linda.scarrott@boehringer-ingelheim.com (L.O.); c.hille@uke.de (C.H.); s.joosse@uke.de (S.A.J.); s.riethdorf@uke.de (S.R.); pantel@uke.de (K.P.); 3 Institute of Hematopathology Hamburg HpH, 22547 Hamburg, Germany; schatz@hp-hamburg.de (S.S.); falk@hp-hamburg.de (M.F.); mtiemann@hp-hamburg.de (M.T.); 4 Boehringer Ingelheim Pharma GmbH & Co. KG, 88397 Biberach, Germany; 5 Institute of Pathology, University Medical Center Hamburg-Eppendorf, 20246 Hamburg, Germany; r.simon@uke.de; 6 Lung Clinic Grosshansdorf, Airway Research Center North, German Center of Lung Research, 22927 Grosshansdorf, Germany; M.Reck@lungenclinic.de

**Keywords:** NSCLC, circulating tumor cells, PD-1/PD-L1 inhibition, resistance

## Abstract

Circulating tumor cells (CTCs) hold great potential to answer key questions of how non-small cell lung cancer (NSCLC) evolves and develops resistance upon anti-PD-1/PD-L1 treatment. Currently, their clinical utility in NSCLC is compromised by a low detection rate with the established, Food and Drug Administration (FDA)-approved, EpCAM-based CellSearch^®^ System. We tested an epitope-independent method (Parsortix^TM^ system) and utilized it to assess PD-L1 expression of CTCs from NSCLC patients. We prospectively collected 127 samples, 97 of which were analyzed with the epitope-independent system in comparison to the CellSearch system. CTCs were determined by immunocytochemistry as intact, nucleated, CD45^−^, pankeratins (K)^+^ cells. PD-L1 status of CTCs was evaluated from 89 samples. With the epitope-independent system, ≥1 CTC per blood sample was detected in 59 samples (61%) compared to 31 samples (32%) with the EpCAM-based system. Upon PD-L1 staining, 47% of patients harbored only PD-L1^+^CTCs, 47% had PD-L1^+^ and PD-L1^−^CTCs, and only 7% displayed exclusively PD-L1^−^CTCs. The percentage of PD-L1^+^CTCs did not correlate with the percentage of PD-L1^+^ in biopsies determined by immunohistochemistry (*p* = 0.179). Upon disease progression, all patients showed an increase in PD-L1^+^CTCs, while no change or a decrease in PD-L1^+^CTCs was observed in responding patients (*n* = 11; *p* = 0.001). Our data show a considerable heterogeneity in the PD-L1 status of CTCs from NSCLC patients. An increase of PD-L1^+^CTCs holds potential to predict resistance to PD-1/PD-L1 inhibitors.

## 1. Introduction

Blockade of the interaction of programmed death ligand 1 (PD-L1) and its receptor PD-1 has revolutionized treatment of patients with non-small cell lung cancer (NSCLC) [1]. Therapy with different anti-PD-1/PD-L1 antibodies was approved in NSCLC in the first- and second-line setting. A correlation between PD-L1 expression by tumor or immune cells and efficacy of anti-PD-1/PD-L1 antibodies has been confirmed in various trials. However, the predictive valus of PD-L1 immunohistochemistry (IHC) is still controversial since activity has also been observed in patients with PD-L1-negative tumors [2,3]. One possible explanation for these findings could be that a single tissue biopsy does not adequately reflect the heterogeneity of PD-L1 expression in stage IV NSCLC patients with multiple tumor sites. Furthermore, dynamic changes of PD-L1 expression in tumor cells might occur before or under therapy with PD-1/PD-L1 inhibitors, leading to different sensitivity to PD-1/PD-L1 blockade which would similarly be missed by one single biopsy.

Circulating tumor cells (CTCs) hold promise to better reflect the tumor heterogeneity compared to tissue biopsies because they originate from different tumor sites. In addition, they could lead to important insights on how tumor cells become resistant to immune therapy because they can be analyzed longitudinally as liquid biopsies [4].

One major challenge remains the specific detection of CTCs in cancer patients. The semiautomated, EpCAM/pankeratins(K)-dependent CellSearch^®^ system is the only CTC detection method approved by the Food and Drug administration (FDA). While this system proved clinical utility in breast, prostate, and colorectal cancer, its usefulness in NSCLC remains challenging [5]. Few reports suggest that in NSCLC, one or more CTCs can be detected by CellSearch in only 30% of patients compared to 50% and 57% of patients with more than five CTCs in breast and prostate cancer, respectively [6,7]. Thus, alternative methods for CTC detection are warranted, especially in NSCLC, in order to allow clinically meaningful applications in a representative number of patients. Especially based on recent reports on CTCs with lower EpCAM expression and increased CTC heterogeneity, one strategy is to use label-independent, microfluidic devices [8,9].

In this prospective study, we aim to evaluated the label-independent, microfluidic Parsortix^TM^ system (ANGLE plc., Guildford, United Kingdom), which selects CTCs based on size and rigidity in a cohort of 127 samples form NSCLC patients [10]. To the best of our knowledge, this is the first study using the label-independent Parsortix system for CTC detection in a large cohort of NSCLC patients. We also compared CTC detection directly to the CellSearch system, which is considered the gold-standard, using matched samples. In a second step, we established a workflow to detect PD-L1^+^ CTCs in Parsortix-enriched samples in order to shed light on the largely unanswered question about heterogeneity of PD-L1 expression by CTCs in NSCLC. Furthermore, we assessed the PD-L1 expression by CTCs under therapy with anti-PD-L1/PD-1 antibodies.

## 2. Results

### 2.1. CTC Enumeration

**Figure 1:**
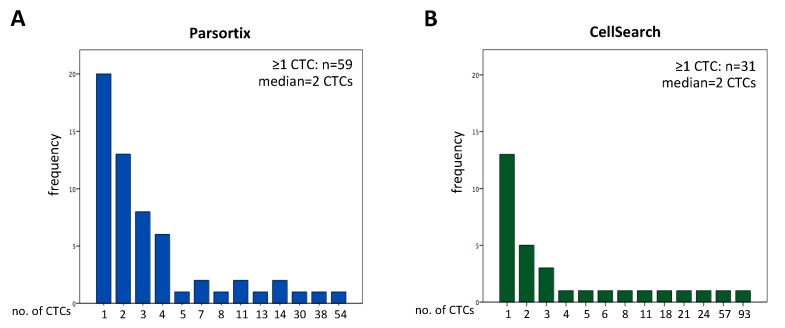
Distribution of CTCs detected with the label-independent Parsortix system (**A**) and with the EpCAM-based CellSearch system (**B**). *n* = 97 total samples from the matched cohort (Table 1) were analyzed in parallel.

**Figure 2:**
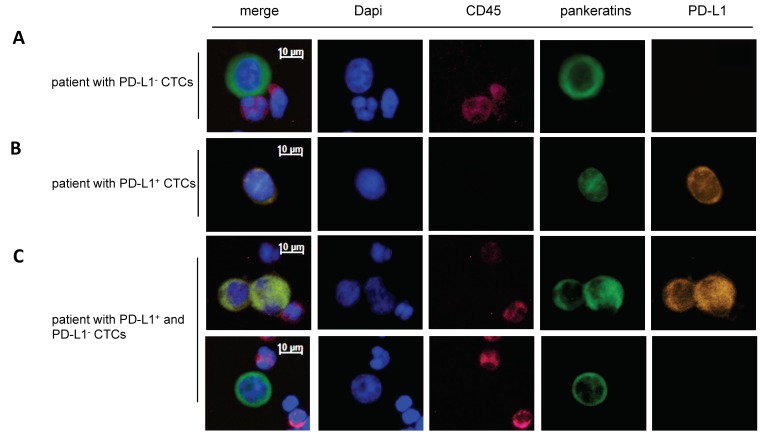
Representative images of CTCs detected with the Parsortix system. Cells were subjected to immunostaining with DAPI, CD45, pankeratins (K), and PD-L1 (D8T4X) after Parsortix enrichment. Representative images of CTCs from patients with only PD-L1^−^ (**A**), only PD-L1^+^ (**B**) and both PD-L1^−^ and PD-L1^+^ CTCs are shown (**C**). The scale bar of 10 µm applies to all pictures.

With the label-independent Parsortix system, we detected ≥1 CD45^−^/K^+^ cell in 59 and ≥3 CD45^−^/K^+^ cells in 26 out of 97 samples (60.8% and 26.8%, respectively, range: 1–54 CTCs, median = 2, Table 1, matched cohort and Figure 1A). Figure 2 shows representative images of CD45^−^/K^+^ nucleated cells detected with the Parsortix system.

In order to confirm the identity of CD45^−^/K^+^ cells detected by the Parsortix system, we isolated them by micromanipulation and performed Sanger sequencing on KRAS after whole genome amplification (WGA) of single cell DNA. Thereby, we found the same KRAS-G12C mutation (c.34G>T) detected in the tumor biopsy of one patient at initial diagnosis also in the DNA from six CTCs isolated from that patient by the microfluidic system. In NSCLC, KRAS-mutations mostly occur in codon 12 and are almost exclusively found in patients with adenocarcinomas [11].

Compared to the label-independent method, we found ≥1 CTC in only 31.9% (*n* = 31) and ≥3 CTCs in 13.4% (*n* = 13) of the samples (range: 1–93 CTCs, median = 2; Figure 1B) by using the EpCAM-based CellSearch. This detection rate for CTCs in NSCLC patients with the CellSearch system is in line with other NSCLC studies reporting a detection rate of 21–39% for ≥1 CTC per blood sample [12,13]. In 37 out of 97 samples, ≥1 CTC was detected with the label-independent Parsortix system only, in 20 patients with both system and in 11 out 97 samples CTCs were found only with the EpCAM based CellSearch system and not with the Parsortix device. Based on the CTC status of the matched samples, the McNemar’s exact test (*p* < 0.001) showed no agreement between the two systems indicating that after enrichment with the Parsortix system ≥1 CTC was detected in significantly more patients compared to the CellSearch system.

Of note, 49 of the 97 samples were analyzed with the CellSearch CXC kit, while the rest was analyzed with the CellSearch CTC kit (both from Menarini Silicon Biosystems, Florence, Italy). The kits differ by the fluorochromes conjugated to pankeratin, which has been discussed to lead to differences in CTC detection [14]. We detected CTCs in 13 out of 49 patients (26.5%, range 1–21) using the CXC kit, while we found CTCs in 18 out of 48 samples (37.5%, range 1–93) using the CTC kit. In our cohort, the CTC detection rate is lower using the CXC kit. However, the detection rates with both CellSearch kits were still significantly lower compared to the detection rate obtained with the Parsortix system (*p* = 0.002 for ≥1 CTC with Parsortix vs. CXC, *p* = 0.027 for ≥1 CTC with Parsortix vs. CTC, McNemar’s Exact test).

### 2.2. Identification of Pankeratin^+^ Cell Cluster

Additionally, we found K^+^ cell clusters in 10.3% of the samples with the label-independent device (10/97 samples, 1–3 clusters, Figure S1). It remains unclear if these clusters are indeed CTC clusters since genomic analyses and further phenotypic characterization is missing. Knowledge about CTC clustering is still sparse but they have been reported to have a higher metastatic potential and are associated with a poorer outcome [15]. Two recent studies reported that CTC clusters show specific changes in DNA methylation that promote stemness and metastasis and that neutrophils accompanying CTC clusters also promote an enhanced metastatic capacity by inducing cell cycle progression [16,17]. Notably, in one of the patients in this cohort, all three clusters were accompanied by CD45^+^ cells, most likely to be identified as neutrophils based on their segmented chromatin. One group reported a frequency of CTC clusters in lung cancer similar to that in our cohort. In this study, CTC clusters were detected in 4 out 33 samples [18]. Interestingly however, in this cohort, 30% of the patients had early stage lung cancer (stage I and II). By using a straight microfluidic size-based microchip in head and neck cancer, the same group found CTC clusters in nine out 21 patients, three of which also had stage I or II disease.

### 2.3. PD-L1 Status of CTCs in NSCLC Patients

We developed a PD-L1 staining protocol for tumor cells enriched by the Parsortix system by spiking PD-L1^+^ H1975 vs. PD-L1^−^ MCF7 cells in whole blood obtained from healthy donors. The PD-L1 status of both cell lines was known from the literature and was confirmed by RT-PCR and Western blot analyses (Figure S2) [19,20].

**Figure 3:**
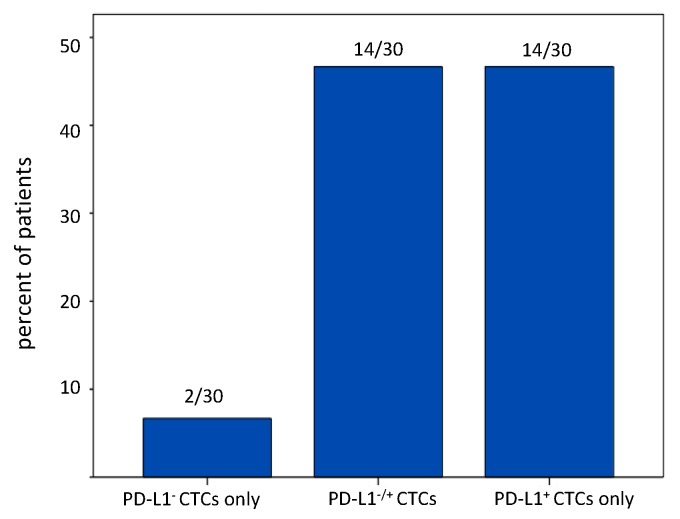
PD-L1 expression by CTCs detected with the Parsortix system. Percentage of patients with only PD-L1^−^, only PD-L1^+^ and with both types of CTCs detected with Parsortix. Only patients with ≥3 CTCs were included (30 out of 89 patients, PD-L1 cohort, Table 1). The numbers on top of each bar indicate the actual number of patients with only PD-L1^−^, only PD-L1^+^ and with both PD-L1^−^ and PD-L1^+^ CTCs.

We investigated the PD-L1 status of CTCs enriched by the label-independent Parsortix system in 89 samples (for patients’ characteristics see Table 1, “PD-L1 cohort”). In the PD-L1 cohort, the CTC-detection rate was similar to the matched cohort that was used for the comparison of the label-independent method with the EpCAM-based isolation: ≥1 CTC in 61/89 samples (68.5%) and ≥3 CTCs in 30/89 samples (33.7%). We detected ≥1 PD-L1^+^ CTC in 50 out of 89 (56%) and ≥3 PD-L1^+^ CTCs in 23 out of 89 (26%) samples. Amongst patient samples with at least three CTCs (CD45^−^/K^+^, 30/89), 47% (14/30) harbored exclusively PD-L1^+^ CTCs and 47% (14/23) had both PD-L1^+^ and PD-L1^−^ CTCs. Interestingly, only two patients displayed exclusively PD-L1^−^ CTCs (7%, 2/30) (Figure 2A–C and Figure 3).

Altogether, there is a considerable heterogeneity of PD-L1 expression in CTCs of NSCLC patients.

### 2.4. Comparison of PD-L1 Status of CTCs Detected with the Parsortix System in Comparison to the PD-L1 Status in Tumor Biopsies 

**Figure 4:**
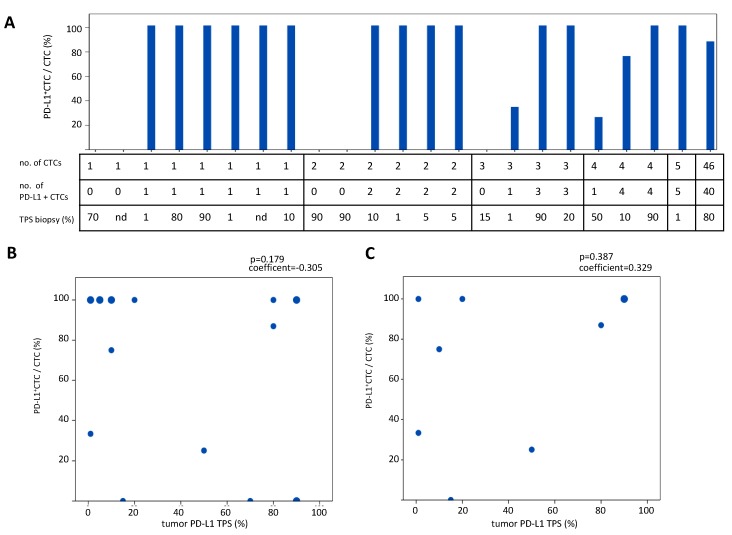
Correlation of PD-L1 expression in CTCs with PD-L1 tumor proportional score (TPS). (**A**) Schematic overview of percentage of PD-L1^+^CTCs in *n* = 23 patients with ≥1 CTC at initial diagnosis before start of any treatment compared to percentage of PD-L1^+^ tumor cells in the primary biopsy (TPS; IHC). (**B**) Correlation of percentage of PD-L1^+^CTCs at initial diagnosis with PD-L1 TPS from biopsies in patients with ≥1 CTCs (*n* = 23, Spearman’s correlation). (**C**) Correlation of percentage of PD-L1^+^CTCs at initial diagnosis with PD-L1 TPS in patients with ≥3 CTCs (*n* = 9, Spearman’s correlation). The larger data points consist of data from more than one patient (**B**,**C**).

We obtained samples from 36 patients at initial diagnosis in 23 of which one or more CTCs were detected. We compared the PD-L1 status of CTCs in these 23 samples with the PD-L1 expression in the primary tumor biopsy at initial diagnosis (tumor proportional score, TPS, Figure 4A). We found that the PD-L1 TPS did not correlate with the percentage of PD-L1^+^ CTCs at initial diagnosis (*p* = 0.179, spearman correlation, *n* = 23/36, 63.8% with ≥1 CTC and *p* = 0.387, spearman correlation, *n* = 9/36, 25% with ≥3 CTCs, Figure 4A–C). We also analyzed the distribution of PD-L1^+^ CTCs in patients harboring ≥3 PD-L1^+^ CTCs at initial diagnosis because it is difficult to calculate percentages in patients with extremely low numbers of CTCs and results need to be interpreted with caution. Amongst the patients with ≥3 CTCs (*n* = 9), we observed that 60% of patients with a PD-L1 TPS < 50% (*n* = 5) had more than 50% PD-L1^+^ CTCs expressing at initial diagnosis (three out of five patients with PD-L1 TPS >50% and ≥3 CTC) (Figure 4A). Thus, the PD-L1 status of CTCs does not correlate with the PD-L1 TPS.

### 2.5. Evaluation of PD-L1 Status of CTCs upon PD-1/PD-L1 Blockade

**Figure 5:**
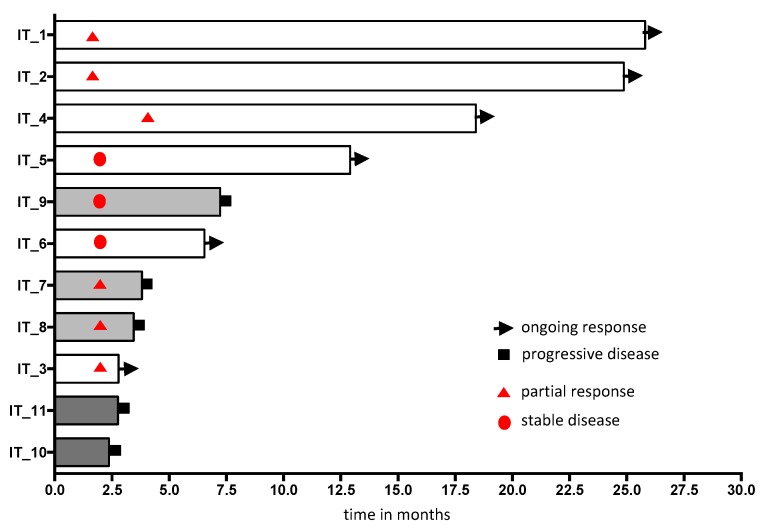
Progression free survival for patients with longitudinal samples of expression of PD-L1 in CTCs. White bar, ongoing response; light grey bar, evasive resistance; grey bar, primary resistance.

We prospectively collected longitudinal samples of 11 patients undergoing therapy with pembrolizumab, nivolumab or atezolizumab. Of those, five patients were treated first-line with pembrolizumab, while the rest was treated in second or subsequent lines of therapy (Table 2). We collected a pretreatment sample, one sample at the time of first staging (after 3–5 cycles of treatment), and one sample upon progression. We quantified PD-L1^+^ and PD-L1^−^ CTCs at each time point. This strategy allowed us to assess the dynamics of CTC counts and of the fraction of PD-L1^+^ CTCs upon anti-PD-1/PD-L1 treatment. We categorized patients into two groups: those deriving clinical benefit by achieving partial remission or stable disease (no patients achieved complete remission) and those with progressive disease at the first staging or after an initial response. According to these criteria, nine patients were responders, three of which developed evasive resistance and two patients were primarily resistant to PD-1/PD-L1 blockade (Table 2). Figure 5 depicts the progression free survival (defined as time from start of immunotherapy to progression or death).

Our data indicate that 89% of the responding patients exhibited either a decrease or no change of their total CTC counts after three or five cycles of therapy (decrease: 6/9; no change 2/9, increase: 1/9). In contrast, the two primarily resistant patients had an increase of their CTC counts. Upon development of resistance all patients (*n* = 5, two primarily resistant patients and three patients with evasive resistance) showed an increase of CTCs compared to earlier time points (*p* = 0.003, decrease or no change vs. increase of CTCs at response vs. progression, Fisher’s Exact test, Table 2). Interestingly, all responding patients (8/8) showed a decrease (5/8) or no change (3/8) in PD-L1^+^CTCs at the time of response compared to before initiation of anti-PD-1/PD-L1 treatment. In contrast, all patients showed an increase in PD-L1^+^CTCs at progression compared to either initiation of treatment in the two primarily resistant patients or compared to the time point of response in the three patients who initially responded but developed evasive resistance (*p* = 0.001, decrease or no change vs. increase of CTCs at response vs. progression Fisher’s Exact test, Table 2 and Table 3). Interestingly, all CTCs were PD-L1^+^ upon development of resistance (Table 3).

## 3. Discussion

To our knowledge, this is the first study including >100 patient samples for the evaluation of efficacy of the label-independent, microfluidic Parsortix system in NSCLC patients. We directly compared CTCs enumeration with the FDA-approved, EpCAM-based CellSearch system and found an increase in CTC counts with the label-independent method. However, the number of detected CTCs amongst patients with ≥1 CTC was similarly low with both systems (median for both systems: 2 CTCs Figure 1). Additionally, we report a method for PD-L1 staining of Parsortix-enriched CTCs and show that the PD-L1 status of CTCs does not correlate with the PD-L1 TPS, thus indicating considerable heterogeneity between the primary tumor and CTCs (Figure 4). Furthermore, we found that a decrease or no change of PD-L1^+^ CTCs correlated with the response to anti-PD-1/PD-L1 antibodies. Interestingly, our data show that all CTCs are PD-L1^+^ upon development of primary or evasive resistance (Table 2 and Table 3). These findings put forward the hypothesis that CTCs could be a biomarker for resistance to anti-PD-1/PD-L1 antibodies in NSCLC.

Until now, only very scarce data were reported from smaller studies evaluating the label-independent Parsortix system as a promising new device for CTC enrichment [10,16,21,22,23,24]. It is unclear why the overall CTC detection rate in NSCLC patients is increased with the Parsortix compared to the CellSearch system. One hypothesis is that due to the heterogeneity and low EpCAM expression of some CTC, the EpCAM-based CellSearch might fail to detect certain subpopulations, such as EpCAM^low^ CTCs [8,9]. This hypothesis has to be confirmed in future studies because we did not quantify the EpCAM expression level in Parsortix-enriched CTCs. However, for SCLC, this hypothesis was tested by Chudziak et al. [24]. They showed a higher recovery of EpCAM^low^ tumor cell using SCLC cell lines with high and low EpCAM expression and a higher CTC count using the label-independent Parsortix device compared to the EpCAM-based CellSearch system in all 12 patients’ samples [24]. Nevertheless, in a small proportion of samples, we only detected CTCs with the EpCAM-based CellSearch system and not with the label-independent Parsortix system. This argues against the hypothesis that differences between the two enrichment methods are solely based on EpCAM expression. Other studies also showed an increased detection of CTCs with label-independent methods in direct comparison to the EpCAM-based, FDA-approved CellSearch method. Two of these studies used the filter- and size-based ISET (isolation by size of epithelial tumor cells) system [25,26] and one study used a miniaturized microcavitiy array (MCA) [27].

So far, our data indicate that specific CTC enrichment techniques might be warranted according to the phenotypic characteristics of the CTCs. While the CellSearch system is very suitable for some tumor types, including prostate, breast, and colorectal cancer, it has a low sensitivity in stage IV NSCLC patients, which can be improved with label-independent systems such as the Parsortix system.

To date, only few studies have analyzed the PD-L1 expression in CTCs of patients with different solid tumors such as breast cancer [28], head and neck cancer [29,30,31], prostate cancer [32], gastrointestinal cancers [19,32], bladder cancer [33], melanoma [34], Merkel-cell carcinoma [35], and NSCLC [18,20,36,37,38,39,40,41,42,43,44]. Our study is the first analyzing PD-L1 expression in CTCs enriched by the label-independent Parsortix system. In most PD-L1 CTC studies, different enrichment techniques, methods for CTC detection, PD-L1 antibodies, and patient cohorts were used. Therefore, comparisons between these studies have to be interpreted with caution. Nevertheless, in concordance with two recent studies, we found that the expression of PD-L1 in tumor tissue did not correlate with the fraction of PD-L1^+^ CTCs [39,43]. This indicates considerable heterogeneity between primary tumors and CTCs with respect to PD-L1 expression. In contrast, one study reported good agreement between PD-L1 expression in CTCs and matched tumor tissues [38]. However, in the latter study, the SP142 antibody was used, which has a lower sensitivity for PD-L1 detection in tumor cells and only ten out of 71 patients (14%) expressed ≥1% PD-L1 in the tumor tissue, which is far less than the expected 50–60% reported in literature [2,45,46].

Four of the nine responding patients still had PD-L1^+^ CTCs at the time of a first staging and initial response (3rd–5th application). However, their numbers and percentages were decreased compared to the time point before initiation of immunotherapy. This argues against the hypothesis that any existing PD-L1^+^ CTC population may be indicative of resistance. Instead, our data suggest that the dynamic change and increase in PD-L1^+^ CTCs is associated with resistance. However, the numbers are very low and need to be validated in a larger study cohort.

This finding appears counterintuitive at first, because a higher percentage of PD-L1 positive tumor cells in tissue biopsies correlates with response to anti-PD-1/PD-L1 antibodies [3]. However, our finding is in line with data from an earlier study in which, after 6 months of treatment, five out of five NSCLC patients with progressive disease or death had PD-L1^+^ CTCs, whereas none of the responding patients harbored PD-L1^+^ CTCs (*n* = 5, SD and PR) [36]. Additionally, a study including 35 patients with different gastrointestinal tumors revealed that 95% of the patients with progression developed an increase in PD-L1^high^ CTCs (18/19 with progression) [19]. Furthermore, one report showed an upregulation of PD-L1 in tumor tissue upon disease progression [47]. In this study, longitudinal tissue biopsies from melanoma patients treated with PD-1 inhibitors were taken and it was shown that PD-L1 expression in the tumor tissue is increased in patients upon progression, compared to earlier time points at which they were still responding to the treatment. However, the PD-L1 status of CTCs was not determined in this study, thus it is unclear whether the increased PD-L1 expression detected in tumors is accompanied by increased PD-L1^+^ CTCs [47].

Despite additional evidence from the literature supporting our finding that PD-L1^+^ CTCs increase upon development of resistance to PD-1/PD-L1 inhibitors while they remain constant or decrease in responding patients, our findings are derived from small numbers of patients, warranting validation in larger cohorts. The reasons why PD-L1^+^ CTCs increase upon development of resistance to PD-1/PD-L1 inhibitors are currently not understood. In one previous study, a more spindle-like appearance of PD-L1^+^ CTCs was described, leading to the hypothesis that they represent more aggressive CTCs with an EMT-like phenotype [36,48]. A recent study reported evidence that EMT-related pathways induce PD-L1 expression in cancer stem cells [49].

The evaluation of the prognostic or predictive value of CTCs or PD-L1^+^CTC detected with the Parsortix system is beyond the scope of this study; future work with larger cohorts and longer follow-up are needed to address this point.

Our data show that the determination of the PD-L1 status is feasible in CTCs of NSCLC patients. Larger homogenous cohorts, ideally in the context of randomized clinical trials, are warranted to investigate the predictive value of PD-L1^+^ CTCs and their association with prognosis. In order to move PD-L1 status of CTCs forward as a potential biomarker, harmonization and validation studies have to be performed. Similar to the blueprint studies for tumor tissue [50], these investigations should include different PD-L1 antibodies, but also include validation for different CTC-enrichment methods (i.e., label-dependent vs. label-independent). Future studies may also take into account the effect of different levels of PD-L1 expression on CTCs [19]. This will give additional information on the role of PD-L1 on tumor cells during anti-PD-1/PD-L1 treatment.

## 4. Material and Methods

### 4.1. Patient Characteristics

NSCLC patients treated at the University Medical Center Hamburg-Eppendorf and the LungenClinic Grosshansdorf were enrolled between September 2015 and February 2018. All patients gave written informed consent. This study has been conducted according to the Declaration of Helsinki and was approved by the local ethics committee (ethics review board Aerztekammer Hamburg approval number PV5392).

In total, 127 samples from patients with histologically proven NSCLC were included in this study. Blood samples of 7.5 mL were taken and analyzed with the label-independent Parsortix system and the EpCAM-based CellSearch system (Menarini Silicon Biosystems, Florence, Italy).

At the time of sample collection, most patients had metastatic disease (96%, *n* = 122) and five patients had stage III disease. Eighty-seven percent of the patients were diagnosed with adenocarcinoma (*n* = 111). Blood samples were taken at different time points: 55 samples (43.3%) were taken at initial diagnosis, 42 samples (33.1%) after progression from previous palliative therapies and before the start of a new treatment regimen, and 30 (23.6%) samples were taken as longitudinal follow-up samples during therapy (Table 1).

In 97 patients, two peripheral blood samples were analyzed to compare CTC enumeration by the label-independent Parsortix system with the EpCAM-based CellSearch System (Table 1, matched cohort).

The PD-L1 status of CTCs was determined in 89 samples enriched with the label-independent device (Table 1, PD-L1 cohort). In this PD-L1 cohort, 36 samples were taken at initial diagnosis. These samples were used for comparison of the PD-L1 tumor proportional score (TPS) in biopsies vs. PD-L1 status of CTCs (Figure 4A). We also collected longitudinal samples from before initiation of immune checkpoint therapy, before the 3rd or 5th application, and at the time of progression from immune checkpoint therapy from 11 patients (Table 2).

### 4.2. Detection of CTCs

Blood samples of 7.5 mL were collected into ethylenediaminetetraacetic acid (EDTA) tubes (Sarsted, Nürnbrecht, Germany) and/or CellSave tubes (Menarini Silicon Biosystems, Florence, Italy). EDTA samples were processed within 24 h on the label-independent, microfluidic system (Parsortix, ANGLE plc., Guildford, United Kingdom) and CellSave samples were analyzed within 96 h on the EpCAM-based CellSearch system.

#### 4.2.1. EpCAM-Based CellSearch System

The CellSearch system is a semiautomated EpCAM/pankeratin-based CTC enrichment technique. It enumerates tumor cells of epithelial origin (CD45^−^, EpCAM^+^ and keratins 8, 18, and 19) in whole blood and was used as described previously [5].

#### 4.2.2. Label-Independent, Microfluidic Parsortix System

The Parsortix cell separation system provides a size- and deformability-based enrichment with the option of subsequent recovery (harvesting) of cells from the device. A capturing cassette with a size-restricted gap of 6.5 µm was used in conjunction with a fluidic processor (ANGLE plc., Guildford United Kingdom). The Parsortix system was used as previously published [10,22]. The microfluidic device allows for antibody-independent cell separation. Separated cells were harvested and spun onto a glass slide (190 g, 5 min). Slides were dried overnight at room temperature and stored at −80 °C until further analysis.

### 4.3. Immunocytochemistry

Following enrichment by the Parsortix system, immunocytochemistry was used to identify CTCs and tumor cells spiked into human blood. Briefly, after fixation with 2% paraformaldehyde (PFA), cells were stained on a glass slide using 4′,6-diamidino-2-phenylindole (DAPI, Janssen Diagnostics, Raritan, NJ, USA; 1:1000), pankeratins (AE1/AE3, 53-9003-82, eBioscience, Waltham, MA, USA) and C11, #4523, Cell Signaling Technology, CST, San Diego, CA, USA 1:300 each), CD45 (HI30, # 304018, BioLegend, Beverly, MA, USA, 1:200) and PD-L1 (D84TX, #86744, CST, San Diego, CA, USA, 1:50). After staining, the cells were washed with phosphate buffered saline, covered with a coverslip and examined by fluorescence microscopy. Intact, nucleated CD45^−^/K^+^ cells were interpreted as CTCs.

The PD-L1 staining was established using spiked human blood samples. A Ficoll gradient was performed from 7.5 mL blood samples taken from healthy individuals at the Transfusionsmedizin (UKE, Germany). The mononucleated layer was collected and subsequently spiked with MCF7 cells (K^+^/PD-L1^−^) or H1975 cells (K^+^/PD-L1^+^). The spiked samples were spun on a glass slide by centrifugation. The generated slides were stained as described above to develop a specific staining protocol. Complete absence of staining was regarded as negative for PD-L1.

### 4.4. Cell Lines

The origin of the cell lines was verified by Multiplex human cell line authentication test at Multipexion GmbH (Friedrichshafen, Germany). Cells were cultured under standard conditions in humidified incubators at 37 °C with 5% CO_2_ in DMEM (MCF7) or RMPI (H1975). The media were supplemented with 10% fetal calf serum and 1% penicillin/streptomycin. All cell culture reagents were purchased from Invitrogen, Darmstadt, Germany.

### 4.5. Sanger Sequencing of DNA Isolated from CTCs Identified with Parsortix

Target CD45^−^/K^+^ nucleated cells were picked by micromanipulation (micro injector CellTramVario and micromanipulator TransferManNKII, Eppendorf Instruments, Hamburg, Germany). Whole genome amplification (WGA) was performed with the Ampli1^TM^ WGA kit according to the manufacturer’s instructions (Menarini Silicon Biosystems, Florence, Italy). The quality of the WGA product was assessed with the Ampli1™ QC Kit (Menarini Silicon Biosystems, Florence, Italy). KRAS Exon2 and 3 mutational analyses was performed using direct Sanger sequencing (PCR Primers: FOR: 5′TATAAGGCCTGCTGAAAATGAC; REV: 5′TTGTTGGATCATATTCGTCCAC; sequencing primers FOR: 5′GCCTGCTGAAAATGACTG; REV: CGTCCACAAAATGATTCTG).

### 4.6. Staining of Tumor Tissue

Freshly cut tissue sections were immunostained in a DAKO Link 48 autostainer device. Slides were deparaffinized and exposed to heat-induced antigen retrieval for 10 min at 98 °C in pH 9 Tris-EDTA-Citrate buffer. Primary antibody specific for PD-L1 (E1L3N, CST, San Diego, CA, USA, 1:200) was applied at room temperature for 20 min. Bound antibody was then visualized using the EnVision Flex Kit (DAKO, Glostrup, Denmark) according to the manufacturer’s directions. 

Complete absence of staining was regarded as “negative”. Staining of any intensity found in more than 1% of tumor cells was considered positive and the percentage of PD-L1^+^ tumor cells was assessed as described [51].

### 4.7. Statistics

Depending on scale, patient characteristics and results are presented as means, medians, and ranges or counts and percentages. Differences in CTC detection rates between the CellSearch system and the Parsortix system were analyzed using McNemars Exact test. Spearman correlations were used for association of PD-L1 TPS and PD-L1 status of CTCs. A Fisher’s Exact test was used for the comparison of change of PD-L1^+^ CTCs in responding vs. nonresponding patients during treatment with anti-PD-L1/PD-1 antibodies. Statistical significance was defined as *p* < 0.05. Statistical analysis was performed using SPSS Statistics 20.0 (IBM, Armonk, NY, USA).

## 5. Conclusions

Here, we show an improved sensitivity for CTC detection by using a novel, label-independent, microfluidic device for CTC enrichment in NSCLC patients. Notably, the detection of CD45^−^/pankeratin^+^ clusters was possible. We also showed that monitoring of PD-L1 status in CTCs in longitudinal samples throughout therapy is possible. These data revealed that a dynamic increase in PD-L1^+^ CTCs might indicate resistance towards PD-1/PD-L1 inhibitors.

## Figures and Tables

**Table 1:** Patients characteristics. Total cohort, all samples subjected to either the CellSearch or the Parsortix system; matched cohort, sample included in the comparison between the CellSearch and the Parsortix system for circulating tumor cell (CTC) detection in non-small cell lung cancer (NSCLC) patients; programmed death ligand 1 (PD-L1) cohort, PD-L1 cohort, samples for which the PD-L1 status of CTCs was analyzed with the Parsortix system.

Patient Characteristics	Total Cohort (*n* = 127) *n* (%)	Matched Cohort, (*n* = 97) *n* (%)	PD-L1 Cohort, (*n* = 89) *n* (%)
age in years (range)	64.5 (32–83)	63.9 (44–81)	64.9 (32–82)
gender			
female	57 (44.9)	43 (44.3)	38 (42.5)
male	70 (55.1)	54 (55.7)	51 (57.3)
histology			
squamous	15 (11.8)	13 (13.4)	11 (12.4)
adeno	111 (87.4)	83 (85.6)	77 (86.5)
NSCLC-NOS	1 (0.8)	1 (1.0)	1 (1.1)
UICC at diagnosis			
IIB	2 (1.6)*	0	2 (2.2)
IIIA	5 (3.9)	5 (5.2)	1 (1.1)
IIIB	11 (8.7)	9 (9.3)	9 (10.1)
IIIC	6 (4.7)	3 (3.1)	6 (6.7)
IV	103 (81.1)	80 (82.5)	71 (79.8)
Sample taken at			
initial diagnosis	55 (43.5)	45 (46.4)	36 (40.4)
progression from previous palliative therapy	42 (33.1)	34 (35.1)	27 (29.2)
during treatment	30 (23.6)	18 (18.6)	26 (29.2)
Treatments			
chemotherapy	41 (32.3)	35 (36.1)	21 (23.6)
immune checkpoint inhibitor	48 (37.8)	40 (41.2)	44 (49.4)
targeted therapy	24 (18.9)	13 (10.2)	15 (16.9)
chemotherapy + surgery +/− radiotherapy (oligometastic)	4 (3.1)	2 (2.1)	4 (4.5)
combined radiochemotherapy	2 (1.6)	1 (1.0)	2 (2.2)
surgery	1 (0.8)	1 (1.0)	0
best supportive care	4 (3.1)	2 (2.1)	3 (3.4)
missing	3 (2.4)	3 (3.1)	0

**Table 2:** Longitudinal analysis of expression of PD-L1 in CTCs. Tabular overview of CTCs and PD-L1^+^CTCs during treatment with anti-PD-1/PD-L1 therapies. Samples were taken before initiation of treatment, after three to five applications and at progression. White background, ongoing response; light grey background, evasive resistance; dark grey background, primary resistance; na, not applicable; SD, stable disease, PR, partial remission.

ID	Line	Drug	CTC Classification	CTC Numbers			Best Response
				start	#3–5	PD	
**IT_1**	1st line	pembrolizumab	total CTCs	2	0	na	PR
			PDL1^+^CTCs	0	0		
**IT_2**	1st line	pembrolizumab	total CTCs	0	2	na	PR
			PDL1^+^CTCs	0	0		
**IT_3**	2nd line	atezolizumab	total CTCs	11	2	na	PR
			PDL1^+^CTCs	10	2		
**IT_4**	1st line	pembrolizumab	total CTCs	4	4	na	PR
			PDL1^+^CTCs	4	4		
**IT_5**	2nd line	nivolumab	total CTCs	4	1	na	SD
			PDL1^+^CTCs	4	1		
**IT_6**	2nd line	nivolumab	total CTCs	3	1	na	SD
			PDL1^+^CTCs	3	1		
**IT_7**	1st line	pembrolizumab	total CTCs	0	0	1	PR
			PDL1^+^CTCs	0	na	1	
**IT_8**	2nd line	nivolumab	total CTCs	2	0	1	PR
			PDL1^+^CTCs	1	0	1	
**IT_9**	3rd line	nivolumab	total CTCs	4	0	2	SD
			PDL1^+^CTCs	2	0	2	
**IT_10**	2nd line	pembrolizumab	total CTCs	0	3	3	na
			PDL1^+^CTCs	0	3	3	
**IT_11**	1st line	pembrolizumab	total CTCs	0	14	14	na
			PDL1^+^CTCs	0	14	14	

**Table 3:** Based on Table 2, this table shows the number of patients with a decrease/no change or increase in PD-L1^+^ CTCs at first response (five with partial remission, three with stable disease) compared to initial diagnosis (responding patients) and the number of patients with a decrease/no change or increase in PD-L1^+^ CTCs amongst patients with primary (*n* = 2) or evasive resistance (*n* = 3) compared to initial diagnosis or response (nonresponding/resistant patients).

Patients	Number of Patients with a	
	Decrease /No Change of PD-L1^+^ CTCs	Increase of PD-L1^+^ CTCs
Responding patients	5 (decrease), 3 (no change)	0
Nonresponding/resistant patients	0	5

## Supplementary Materials

The following are available online at https://www.mdpi.com/2072-6694/11/6/835/s1, Figure S1: Cell cluster identified with label-independent system, Figure S2: Establishing immunocytochemistry for PD-L1 on CTCs.
