# Supplementary material for: Determination of PD-L1 Expression in Circulating Tumor Cells of NSCLC Patients and Correlation with Response to PD-1/PD-L1 Inhibitors
Source: Cancers (Basel). 2019 Jun 17;11(6):835. doi: 10.3390/cancers11060835 (PMC6627043; doi:10.3390/cancers11060835)
Supplement: Supplementary file 1 [file cancers-11-00835-s001.pdf]

Supplementary Material: Determination of PD-L1 Expression in Circulating Tumor Cells of NSCLC Patients and Correlation with Response to PD-1/PD-L1 Inhibitors

Melanie Janning, Franca Kobus, Anna Babayan, Harriet Wikman, Janna-Lisa Velthaus, Sonja Bergmann, Stefanie Schatz, Markus Falk, Lars-Arne Berger, Lisa-Marie Böttcher, Sarina Päsler, Tobias M. Gorges, Linda O'Flaherty, Claudia Hille, Simon A. Joosse, Ronald Simon, Markus Tiemann, Carsten Bokemeyer, Martin Reck, Sabine Riethdorf, Klaus Pantel and Sonja Loges

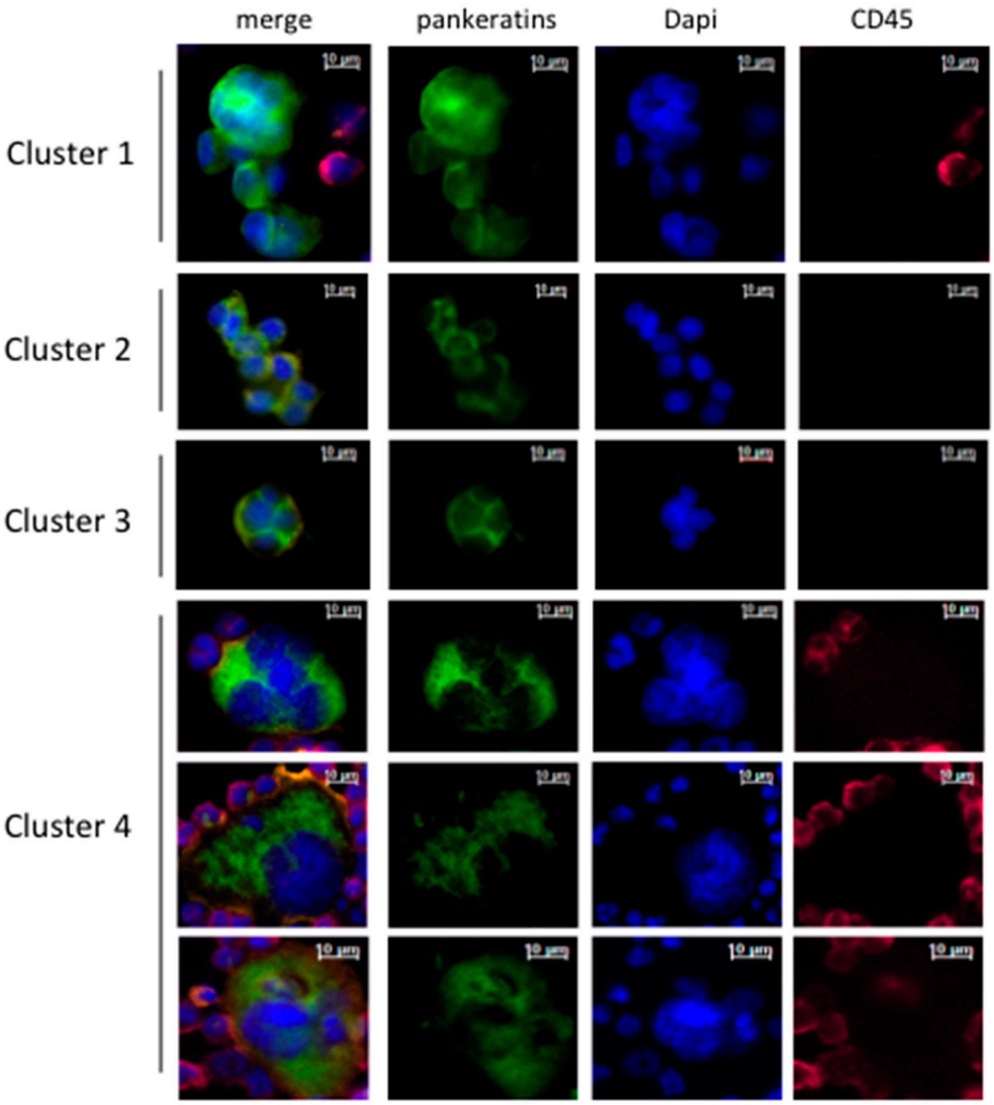

Figure S1. Cell cluster identified with label-independent system. Representative images of cell cluster identified by using the label-independent Parsortix system and immunocytochemistry staining with DAPI, anti-pankeratin (AE1/AE3 and C11) and anti-CD45.

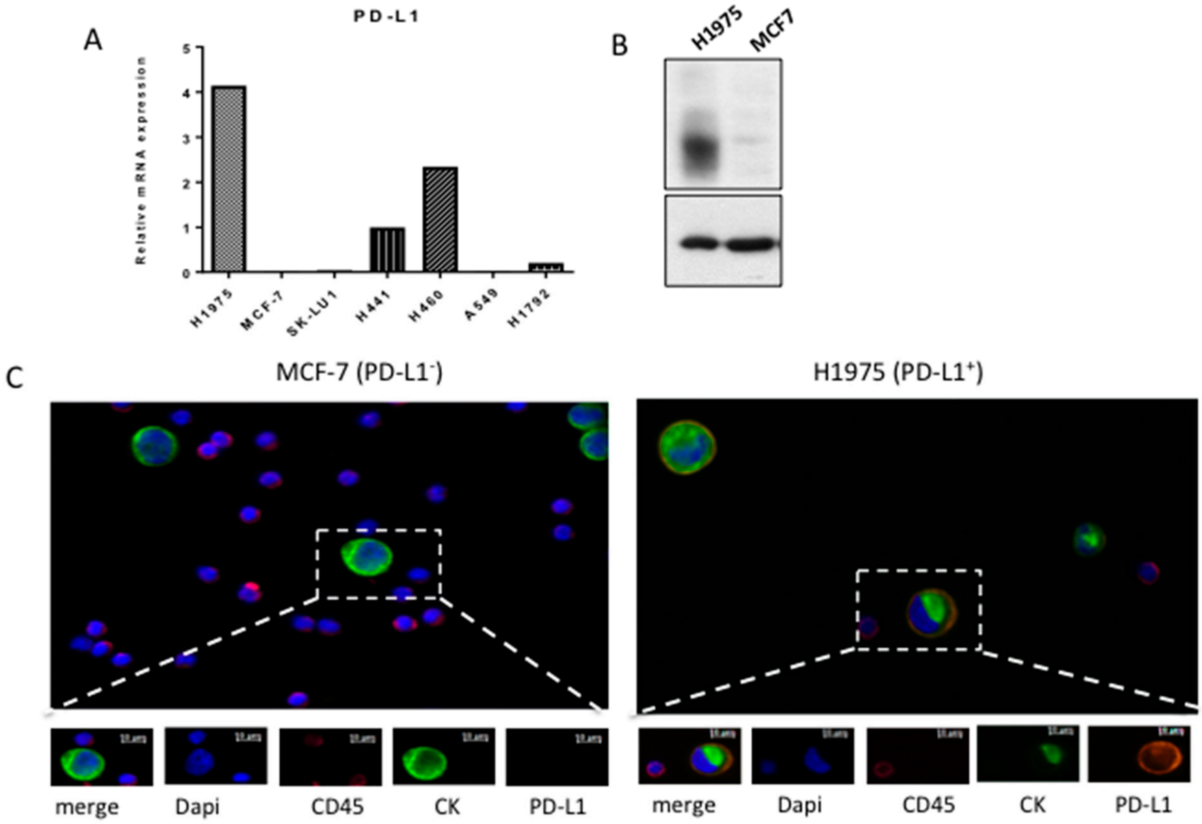

Figure S2. Establishing immunocytochemisty for PD-L1 expression by CTCs. (**A**) PD-L1 RNA transcript level adjusted for GAPDH expression were analyzed from different human cells lines with SyBr green qRT-PCR using primer AGTCAATGCCCCATACAACAAA and TGATGGTCACTGCTTGTCCAG for PD-L1. (**B**) PD-L1 protein level in different human cancer cell lines using anti-PD-L1 (E1L3N). (**C**) Immunocytochemistry staining with DAPI, anti-pankeratin (AE1/AE3 and C11), anti-CD45 and anti-PD-L1 (D8T4X) of H1965 and MCF7 cells spiked in mononucleated cells separated by Ficoll gradient from healthy individuals.

© 2019 by the authors. Licensee MDPI, Basel, Switzerland. This article is an open access article distributed under the terms and conditions of the Creative Commons Attribution (CC BY) license (http://creativecommons.org/licenses/by/4.0/).
